# Reconstruction of enterprise debt networks based on compressed sensing

**DOI:** 10.1038/s41598-023-29595-9

**Published:** 2023-02-13

**Authors:** Kaihao Liang, Shuliang Li, Wenfeng Zhang, Chengfeng Lin

**Affiliations:** 1 Department of Mathematics, Zhongkai University of Agriculture and Engineering, Guangzhou, 510225 China; 2 College of Economics and Trade, Zhongkai University of Agriculture and Engineering, Guangzhou, 510225 China; 3 Institute of Rural Development, Zhongkai University of Agriculture and Engineering, Guangzhou, 510225 China; 4 School of Mathematics and Data Science, Zhongkai University of Agriculture and Engineering, Guangzhou, 510225 China

**Keywords:** Applied mathematics, Computational science, Scientific data

## Abstract

This study aims at the problem of reconstruction the unknown links in debt networks among enterprises. We use the topological matrix of the enterprise debt network as the object of reconstruction and use the time series data of accounts receivable and payable as input and output information in the debt network to establish an underdetermined linear system about the topological matrix of the debt network. We establish an iteratively reweighted least-squares algorithm, which is an algorithm in compressed sensing. This algorithm uses reweighted $\ell _2$-minimization to approximate $\ell _1$-norm of the target vectors. We solve the $\ell _1$-minimization problem of the underdetermined linear system using the iteratively reweighted least-squares algorithm and obtain the reconstructed topological matrix of the debt network. Simulation experiments show that the topology matrix reconstruction method of enterprise debt networks based on compressed sensing can reconstruct over 70% of the unknown network links, and the error is controlled within 2%.

## Introduction

Risk contagion refers to the phenomenon in which an emergency affects the business of related enterprises and then spreads to other enterprises or markets, and it may result in a deepening of risk hazards^1^. Against the background of increasingly detailed enterprise operations, the connection between enterprises is closer, and the impact caused by emergencies is more likely to spread among enterprises, resulting in systemic risks in the market. It can be seen that financial risks have the characteristics of potential, systematicness and complexity. Once they break out, they are quickly transmitted among different markets and difficult to control, making the financial system the most vulnerable link in the modern economy^2^.

To analyze the contagion process of default risk in enterprise debt networks, we must first have a clear understanding of the enterprise debt network. A debt network with full links and information is convenient for analyzing the contagion process of default risk. However, owing to the lack of information about debt relationships among enterprises, the topological structure of the debt network of enterprises is incomplete and some links may be missing in the network. This study reconstructs the topological structure of the debt network of enterprises and discover the unknown link of the debt network using a mathematical method based on compressed sensing.

Our main contribution in this study is that we provide a link reconstruction method based on compressed sensing for complex debt networks among companies. This method uses accounts receivable and accounts payable to establish underdetermined linear systems and solves them with an iteratively reweighted least-squares algorithm. The results showed excellent performance.

Notations: An *s*-sparse vector ${\textbf{x}}$ indicates that it has at most *s* nonzero components, and the other components are zero. $\Sigma _s$ denotes the set of all *s*-sparse vectors. Given matrix $\Phi$ in a real setting, $\Phi ^T$ is the transpose of $\Phi$. $F({\textbf{y}})$ denotes the solution space of the linear equations $\Phi {\textbf{x}} = {\textbf{y}}$. For a vector ${\textbf{x}} \in {\mathbb {R}}^n$ and positive integer *s*, $r({\textbf{x}})_s$ denotes the *s*th largest element of the set $\{|x_j|, j=1,2, \ldots ,n\}$. For a weight ${\textbf{w}} \in {\mathbb {R}}^n$, $\left\| {\textbf{x}} \right\| _{\ell _2({\textbf{w}})}$ is the weighted $\ell _2$-norm, that is,

$$\begin{aligned} \left\| {\textbf{x}} \right\| _{\ell _2({\textbf{w}})} =\left( \sum _{j=1}^{n} w_j x^2_j \right) ^{\frac{1}{2}}. \end{aligned}$$

For a matrix ${\textbf{A}}=(a_{ij}) \in {\mathbb {R}}^{n_1 \times n_2}$, its $\ell _1$-norm is defined as

$$\begin{aligned} \Vert {\textbf{A}}\Vert _1 = \sum _{j=1}^{n_2} \sum _{i=1}^{n_1} |a_{ij}|. \end{aligned}$$

## Literature review

At present, research on the construction of financial market networks is mostly based on the correlation of stock returns. Some scholars used the threshold method^3,4^ to construct a complex network of stock financial markets and characterized its properties with the characteristic parameters of the complex network. The threshold method is a relatively simple method for constructing complex networks, and it is also commonly used in many empirical analyses. However, the threshold method relies heavily on the experience of researchers, and subjective factors significantly influence its performance. Other scholars^5,6^ used the minimum spanning tree method to construct a complex network of stock associations. This type of complex network can analyze the association between different stocks much better and reflect the asset-liability relationship of enterprises to some extent, but ignores known information such as accounts receivable and accounts payable. The threshold and minimum spanning tree method construct the enterprise correlation network through the correlation coefficients of logarithmic return rate of stock prices, and the constructed network can analyze the enterprise risk contagion. However, the enterprise correlation network cannot directly reflect the debt relationship between enterprises. In contrast to correlations based on the stock return rate, some studies build complex networks based on the sparseness of complex network topologies. The essence of complex network reconstruction is to predict the unknown links in complex networks. Wang et al.^7^ proposed a link prediction model that considers the power law distribution of the node degrees. The principle of this model to build a network was to make use of the sparseness of the network topology and to predict network links through node clustering coefficients. Mishchenko et al.^8^ applied a Bayesian framework to reveal the structure of a neural network, constructed the least absolute shrinkage and selection operator (LASSO) problem according to the sparseness of the neural network, and solved it using compressed sensing according to the relationship between the LASSO problem and the compressed sensing problem. However, the application of the Bayesian framework requires some prior information; otherwise, this method cannot be applied. Some scholars^9–12^ revealed the network topology by using the sparsity and power law distribution of network links, proposed to reconstruct the unknown links of the network using compressed sensing, and constructed the network topology identification problem as the sparse solution problem of linear systems. Compressive sensing methods are often used to reconstruct complex network topologies with high accuracy, but current research mainly focuses on the link prediction of physical networks, and the effect of link prediction of complex networks of enterprise debt is unknown.

In addition to signal and image processing, compressed sensing can also be applied to complex network reconstruction of the financial market. By fully considering accounts receivable and accounts payable, the compressed sensing method objectively and accurately reconstructs the debt network of enterprises.

## Reconstruction model of enterprise debt network

**Figure 1:**
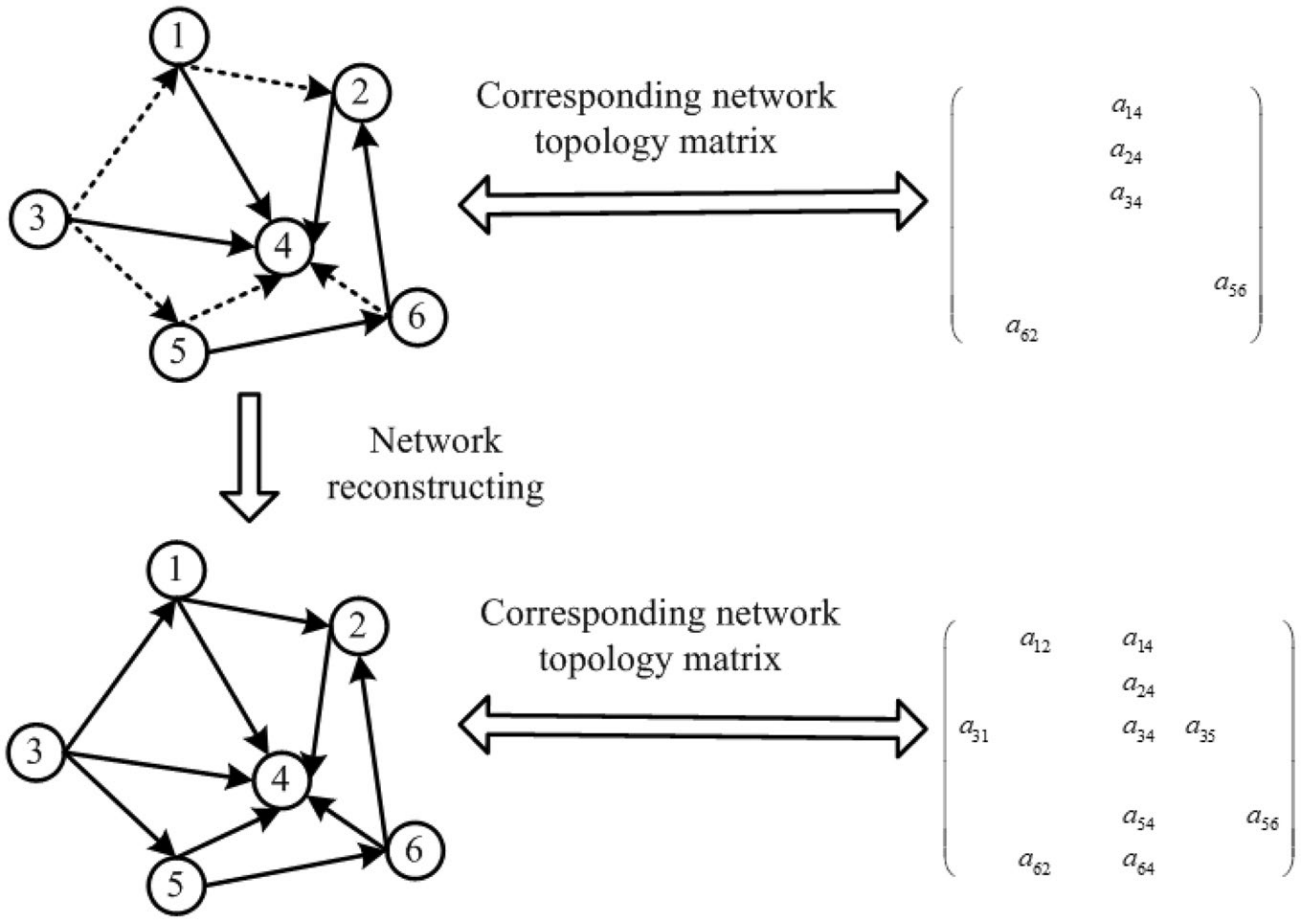
Reconstruction procedure for enterprise debt network.

To analyze the risk contagion and default compensation of companies, it is necessary to accurately construct a debt network among these enterprises. Because of the incompleteness of the information released by these enterprises, it is theoretically difficult to construct a high-precision debt network among these enterprises. In a corporate debt network, an enterprise usually only has debt relationships with a few other enterprises, and the column vectors of the link matrix are sparse, which provides possibility for the debt network reconstruction by compressed sensing^13^. Si et al. used compressed sensing to construct a complex financial network and improved the reconstruction accuracy^14,15^. The debt relationships among enterprises can be expressed through a network. Assuming that there are *n* enterprises, the topology structure of the debt network can be expressed as a matrix $n \times n$, as shown in Fig. 1. Because only partial topological relations are available owing to unreleased information, only partial elements are known in this matrix. In Fig. 1, in the upper-left corner of the network, the solid lines represent known network links, the dotted lines represent hidden network links, and the network topology corresponds to the matrix in the upper-right corner. The reconstruction process of debt network topology among enterprises involves determining the complete network topology matrix according to the element-missing matrix of the debt network topology, and determining the implied network links to determine the accurate enterprise debt network topology^16,17^. When the number *n* of enterprises is sufficiently large, the network structure will show complex network properties, such as self-organization, self-similarity and small world. As a complex network, this network has a cluster effect; that is, only some nodes have a high degree, whereas most nodes have a small degree. In the network topology matrix, the cluster effect is characteristic of the sparsity of its column vector. Therefore, compressed sensing can be used to reconstruct the network topology matrix, and we can accurately reconstruct the complete information of the debt complex network using only underdetermined information^18^. According to the reconstructed debt network, we can accurately analyze the contagion process theory of enterprises’ debt defaults.

Suppose that ${\textbf{x}}(t)= (x_1(t), x_2(t),\ldots , x_n(t))^T \in {\mathbb {R}}^n$ is the state of *n* enterprises in the network at time *t*. In a linear network system, the derivative $\dot{{\textbf{x}}}(t)$ of ${\textbf{x}}(t)$ can be expressed as

$$\begin{aligned} \dot{{\textbf{x}}}(t) = {\textbf{A}} {\textbf{x}}(t)+ {\textbf{B}} {\textbf{u}}(t), \end{aligned}$$

where the matrix ${\textbf{A}} \in {\mathbb {R}}^{n \times n}$ represents the network structure between enterprises. Our objective is to reconstruct the matrix ${\textbf{A}}$ using the time series of ${\textbf{x}}(t)$. Matrix ${\textbf{B}} \in {\mathbb {R}}^{n \times m}$ is the input matrix and the network system is controlled by the input vector ${\textbf{u}}(t)=(u_1(t), u_2(t),\ldots , u_m(t))^T$. Because $\dot{{\textbf{x}}}(t)= {\textbf{x}}(t+1)-{\textbf{x}}(t)$, the system is represented by ${\textbf{x}}(t)$ as follows:

$$\begin{aligned} {\textbf{x}}(t+1) = \bar{{\textbf{A}}} {\textbf{x}}(t) + {\textbf{B}} {\textbf{u}}(t), \end{aligned}$$

where $\bar{{\textbf{A}}} = {\textbf{A}}+\textbf{Id}$ and $\textbf{Id}$ is an identity matrix.

To reconstruct the network topology, we take *p* experiments at time $t_1,t_2, \ldots , t_p$ in the series ${\textbf{x}}(t)$ and ${\textbf{u}}(t)$, and ${\textbf{x}}(t_1+1),{\textbf{x}}(t_2+1),\ldots ,{\textbf{x}}(t_p+1)$ are the output vectors after the action of ${\textbf{x}}(t_i)$ and ${\textbf{u}}(t_i)$ for $i=1,2,\ldots ,p$. We define matrices ${\textbf{X}},{\textbf{Y}},{\textbf{U}}$ as follows:

1 $$\begin{aligned} \begin{array}{l} {\textbf{X}}:=[{\textbf{x}}(t_1), {\textbf{x}}(t_2), \ldots , {\textbf{x}}(t_p)], \\ {\textbf{U}}:=[{\textbf{u}}(t_1), {\textbf{u}}(t_2), \ldots , {\textbf{u}}(t_p)],\\ {\textbf{Y}}:=[{\textbf{x}}(t_1+1), {\textbf{x}}(t_2+1), \ldots , {\textbf{x}}(t_p+1)]. \end{array} \end{aligned}$$

Here, ${\textbf{X}},{\textbf{U}},{\textbf{Y}}$ are called state, input vector and output matrices, respectively. Therefore, we have ${\textbf{Y}} = \bar{{\textbf{A}}} {\textbf{X}} +{\textbf{B}} {\textbf{U}}$, which can be written as

2 $$\begin{aligned}{}[{{\textbf{X}}^T}\quad {{\textbf{U}}^T}]\left[ {\begin{array}{*{20}{c}} \bar{{\textbf{A}}}^T\\ {\textbf{B}}^T \end{array}} \right] = {{\textbf{Y}}^T} \end{aligned}$$

Let matrices $\Phi :=[{\textbf{X}}^T \quad {\textbf{U}}^T]$, $$\Theta := \left[ {\begin{array}{*{20}{c}} \bar{{\textbf{A}}}^T\\ {\textbf{B}}^T \end{array}} \right] $$ and we know that $\Phi$ is a $p \times (n+m)$-dimensional matrix, whereas $\Theta$ is a $(n+m) \times n$ matrix.

The sparsity of the column vector is expressed in the topology matrix of the network owing to the clustering effect of the network. In other words, the column vector of ${\textbf{A}}$ is sparse, meaning that the column vectors of $\Theta$ are sparse. We can reconstruct ${\textbf{A}}$ by recovering $\Theta$ column by column. Let $\eta$ be the *k*th column of $\Theta$ and ${\textbf{y}}$ be the *k*th column of ${\textbf{Y}}^T$, we have

3 $$\begin{aligned} \Phi \eta = {\textbf{y}}. \end{aligned}$$

This is an underdetermined linear system, and compressed sensing has a positive effect on solving the sparse solution of this type of underdetermined linear system^19^.

## Compressed sensing and low-rank matrix recovery

### Compressed sensing

Compressed sensing is a method for recovering sparse signals from an underdetermined linear system. A precondition is that the signal is sparse. As the column vector $\eta$ is sparse, the problem of recovering the sparse signal $\eta$ is transformed into the problem of finding the sparsest solution from the underdetermined linear system (3). Assuming that there exists a uniquely sparsest solution ${\hat{\eta }}$ in the linear system (3), the problem can be expressed by the following mathematical model:

4 $$\begin{aligned} {\hat{\eta }}=\textrm{argmin} \left\| \eta \right\| _0: \mathrm {s.t.}\ \Phi \eta ={\textbf{y}}. \end{aligned}$$

Problem (4) is a combinatorial optimization problem^20,21^, which is NP-hard: when the dimension of the column vector $\eta$ is high, the NP-hard problem is difficult to solve using a computer. Therefore, the natural idea is to solve the convex relaxation $\ell _1$-minimization problem of $\ell _0$-minimization (4). The solution of $\ell _1$-minimization is also a sparse solution. Assuming that there is a unique $\ell _1$-norm minimum solution ${\bar{\eta }}$ in the underdetermined linear system (3), the convex relaxation $\ell _1$-minimization problem can be expressed as

5 $$\begin{aligned} {\bar{\eta }}=\textrm{argmin} \left\| \eta \right\| _1: \mathrm {s.t.}\ \Phi \eta ={\textbf{y}}. \end{aligned}$$

The natural question to ask is whether the solution ${\bar{\eta }}$ obtained by the convex relaxation $\ell _1$-minimization problem is the same as the sparsest solution ${\hat{\eta }}$ that we want to obtain? To solve this problem, Candès^22,23^,Donoho^24^, Romberg^25^, Tao^22^, Gribonval^26^ and others successively proposed the null space property (NSP)^24^ and the restricted isometry property (RIP)^22^. Gribonval^26^ proved that any *s*-sparse signal can be accurately recovered by $\ell _1$-minimization if and only if the measurement matrix satisfies the null space property of *s* order. Candès and Tao^22^ proved that if the measurement matrix $\Phi$ satisfied the restricted isometry property and the restricted orthogonal property, the target sparse signal could be recovered by $\ell _1$-minimization problem. Candès^23^ proved that if the measurement matrix $\Phi$ satisfied the restricted isometry property, and the RIC constant $\delta _{2s} \le 0.4142$, the sparse signal could be accurately recovered by $\ell _1$-minimization. Candès, Romberg and Tao^25^ obtained that if the measurement matrix $\Phi$ satisfied the restricted isometry property and the restricted orthogonal property, the $\ell _1$-regularization problem could stably recover the target signal as long as the target signal was sparse enough. Meanwhile, assuming that the measurement matrix $\Phi$ is a Gaussian random matrix, they showed that the target signal can be stably recovered as long as the sparsity of the target signal has the same order as the number of measurements.

Reconstruction of enterprise debt network is an $\ell _0$-minimization problem (4), and we use iteratively reweighted least squares algorithm to solve the $\ell _1$-minimization (5). Restricted isometry property guarantees that the solution to $\ell _1$-minimization (5) is the solution to $\ell _0$-minimization (4). Meanwhile, it is difficult to verify NSP, but it is relatively easy to verify RIP, which has a certain stability to noise.

#### **Definition 1**

Matrix $\Phi \in {\mathbb {R}}^{p \times n}$ is said to satisfy the restricted isometry property of order *s* if there exists a constant $\delta _s \in (0,1)$,inequality

6 $$\begin{aligned} (1-\delta _s)\Vert {\textbf{x}} \Vert _2^2 \le \Vert \Phi {\textbf{x}}\Vert _2^2 \le (1+\delta _s)\Vert {\textbf{x}}\Vert _2^2 \end{aligned}$$

holds for all ${\textbf{x}} \in \Sigma _s$. The smallest $\delta _s$ is the restricted isometry constant (RIC) of order *s*.

The definition of the restricted isometry property of matrix $\Phi$ shows that if the RIC $\delta _s$ is zero, the matrix $\Phi$ is a column-orthogonal matrix; that is, when the upper bound of $\delta _s$ is smaller, the measurement matrix $\Phi$ is closer to the column-orthogonal matrix. In other words, to ensure that the measurement matrix $\Phi$ satisfies the RIP condition, the columns of the matrix $\Phi$ must be approximately orthogonal. When the upper bound of $\delta _s$ is larger, the number of measurement matrices satisfying the RIP conditions is greater, which means that the range of the measurement matrix is larger, and the flexibility of the measurement matrix is better.

### Low-rank matrix recovery

The low-rank approximation of the matrix is used to find the rank-lowest matrix from a linear measurement, which is also called low-rank matrix recovery. The problem of matrix low-rank approximation can be described as follow. A matrix ${\textbf{X}} \in {\mathbb {R}}^{n_1 \times n_2}$ with its rank at most *r* is observed by the measured vector ${\textbf{y}} = \Phi (X) \in {\mathbb {R}}^p$. Linear operator $\Phi$ is a linear mapping from ${\mathbb {R}}^{n_1 \times n_2}$ to ${\mathbb {R}}^p$. Therefore, the mathematical model for finding the rank-lowest matrix from the linear measurement to approximate the target matrix can be expressed as:

7 $$\begin{aligned} \min \textrm{rank}({\textbf{Z}}): \mathrm {s.t.} \Phi ({\textbf{Z}})= {\textbf{y}}. \end{aligned}$$

Similar to the $\ell _0$-minimization problem, the rank-minimization problem (7) is NP-hard. Considering that the convex relaxation $\ell _1$-minimization of $\ell _0$-minimization problem is a good strategy in the case of a vector, we can relax the rank minimization problem (7) to the kernel norm minimization problem:

8 $$\begin{aligned} \min \Vert {\textbf{Z}}\Vert _*: \mathrm {s.t.} \Phi ({\textbf{Z}})= {\textbf{y}}. \end{aligned}$$

Here, the kernel norm is defined as:

$$\begin{aligned} \Vert {\textbf{Z}}\Vert _*:= \sum _{j=1}^{q} \sigma _j({\textbf{Z}}), q:= \min \{n_1,n_2\}, \end{aligned}$$

In other words, the kernel norm is $\ell _1$- norm of the singular value vector $[\sigma _1({\textbf{Z}}), \ldots , \sigma _q ({\textbf{Z}})]^T$ of matrix ${\textbf{Z}}$.

Similar to compressed sensing, the low-rank matrix recovery problem has a restricted isometry property. Its function is to determine the relationship between the minimum solutions of the kernel norm minimization and rank minimization.

#### **Definition 2**

For a linear mapping $\Phi : {\mathbb {R}}^{n_1 \times n_2} \rightarrow {\mathbb {R}}^p$,$r \le q: = \min \{n_1, n_2\}$, the linear operator $\Phi$ is said to satisfy the restricted isometry property of order *r*, if there exists $\delta \ge 0$, such that for all matrices ${\textbf{X}} \in {\mathbb {R}}^{n_1 \times n_2}$ with the rank of most at *r*, the inequality

9 $$\begin{aligned} (1-\delta )\Vert {\textbf{X}} \Vert _F^2 \le \Vert \Phi ({\textbf{X}}) \Vert _2^2 \le (1+\delta )\Vert {\textbf{X}}\Vert _F^2 \end{aligned}$$

holds. The minimum value of $\delta$ is called the restricted isometry constant $\delta _r = \delta _r (\Phi )$.

Using low-rank matrix recovery to reconstruct the network topology matrix is also a method of network link prediction, but this area requires further research.

## Iteratively reweighted least squares algorithm

The restricted isometric property shows that the $\ell _0$-minimization problem (4) for the reconstruction of enterprise debt network can be obtained by solving the $\ell _1$-minimization (5). To solve the $\ell _1$-minimization problem (5), we provide an iteratively reweighted least-squares algorithm with a tight frame. Iteratively reweighted least squares algorithm approximates the $\ell _1$ minimization by using the weighted $\ell _2$-norm minimization, and it has a wide range of applications. Given a tight frame ${\textbf{D}}= \{{\textbf{d}}_1,{\textbf{d}}_2,\ldots , {\textbf{d}}_l\}$, suppose that $\varepsilon >0$ and a weight vector ${\textbf{w}} \in {\mathbb {R}}^l$ are known, where $w_j >0$, $j=1,2,\ldots ,l$. To establish the algorithm, we define

10 $$\begin{aligned} J_{\textbf{D}}({\textbf{z}},{\textbf{w}},\varepsilon ) \triangleq \frac{1}{2}\left[ \sum _{j=1}^{l} w_j \left| {\left\langle {{\textbf{d}}_j,{\textbf{z}}} \right\rangle } \right| ^2 + \sum _{j=1}^{l} (\varepsilon ^2 w_j+w_j^{-1}) \right] . \end{aligned}$$

The algorithm is as follows.
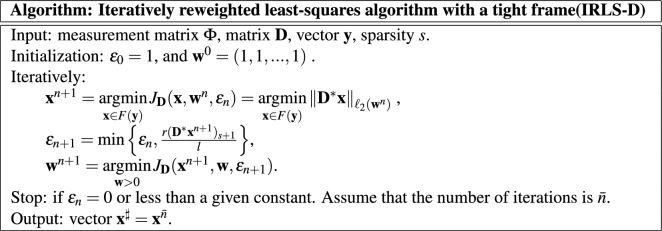


We studied the convergence and robustness of the algorithm^27–29^, and the results showed that the algorithm has good convergence and robustness. Moreover, its convergence rate is high. Iteratively reweighted least-squares algorithm can realize the fast and high-precision reconstruction of enterprise debt network. When this algorithm is applied to solve the $\ell _1$-minimization problem (5), it is only necessary to make tight frame ${\textbf{D}}$ an identity matrix $\textbf{Id}$.

## Numerical simulation

### Reconstruction simulation of enterprise debt network

**Figure 2:**
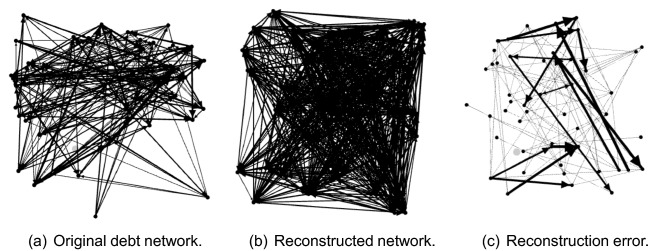
Reconstruction results of debt networks with 50 enterprises. (**a**) Shows the debt relationship before reconstruction, constructed using debt information issued by listed companies. (**b**) Shows the reconstruction result of the debt network by our method based on compressed sensing, whereas (**c**) shows the links that are reconstructed incorrectly, which is the error of reconstruction.

**Figure 3:**
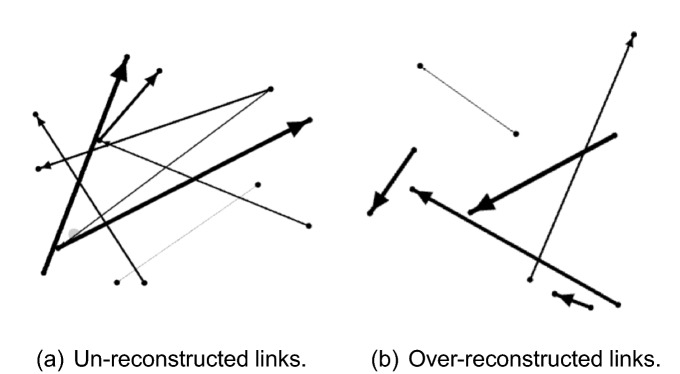
The error between the reconstructed and real network links. (**a**) shows links that are not reconstructed in the real debt network, whereas (**b**) shows the reconstructed links that do not exist in the real debt network.

We considered a debt network with 50 enterprise nodes,and conducted 30 simulation experiments,i.e. $n=50,p=30$. In each simulation experiment, ${\textbf{x}}(t)$ is the debt state vector of 50 enterprises at time *t*, ${\textbf{u}}(t)$ is the accounts receivable of 50 enterprises at time *t*, and ${\textbf{y}}={\textbf{x}}(t+1)$ is the output vector after the action of ${\textbf{x}}(t)$ and ${\textbf{u}}(t)$. We used ${\textbf{x}}(t)$ and ${\textbf{u}}(t)$ to construct the measurement matrix $\Phi$ by formulas (1) and (2). In the process of obtaining ${\textbf{y}}$ by simulation, we add white noise to ${\textbf{y}}$. According to $\Phi$ and ${\textbf{y}}$, topological matrix ${\textbf{A}}$ of the debt network is reconstructed using the iteratively reweighted least-squares algorithm of compressed sensing. In this algorithm, we let ${\textbf{D}}=\textbf{Id}$ and sparsity $s=25$. Figure 2 illustrates the reconstructed enterprise debt network. The simulation results show that there is only a small difference between reconstructed and real debt networks. If $\hat{{\textbf{A}}}$ represents the topological matrix of the reconstructed debt network, and ${\textbf{A}}$ represents the topological matrix of the real debt network, then the error $\Vert \hat{{\textbf{A}}}- {\textbf{A}}\Vert _1/ \Vert {\textbf{A}}\Vert _1$ between them is 1.97%. Compared with the debt network topology matrix ${\textbf{A}}_0$ before reconstruction, we reconstruct 71.36% of the network links in the real debt network; that is, $\Vert \hat{{\textbf{A}}}- {\textbf{A}}_0\Vert _1/ \Vert {\textbf{A}}\Vert _1$ is 71.36%, which means that almost all the hidden links are found. Figure 3 shows the error between the reconstructed network links and real network links. There were two parts to the errors. The first part of the error is the links that have not been reconstructed in the real network, accounting for 1.03% of the real network. The second part of the error is the reconstruction of links that do not exist in the real debt network, accounting for 0.86% of the real network.

### Statistics on network structure characteristics

To analyze the characteristics of the network before and after reconstruction, we imported these two debt networks into Gephi software for data analysis and obtained the network topology characteristics, including the average degree (AD), average weighting degree (AWD), graph density (GD), average clustering coefficient of each node (ACC), and average path length (APL), as shown in Table 1. The results show that compared with the debt network before reconstruction, the four topological features of the reconstructed debt network, such as the average degree, average weighting degree, graph density, and average clustering coefficient of each node, are greatly improved, whereas the average path length is significantly reduced, which indicates that the reconstructed debt network has discovered more hidden network links.

**Table 1:** Network topology characteristics before and after network reconstruction.

Category	AD	AWD	GD	ACC	APL
Reconstructed network	15.74	1.019	0.321	0.313	1.681
Original network	4.84	0.306	0.099	0.099	2.586

## Conclusion

To reconstruct the enterprise debt network to determine its hidden links, we establish $\ell _1$-minimization problem based on compressed sensing and then use the iteratively reweighted least-squares method to solve the problem. The simulation experiments show that the enterprise debt network reconstruction method based on compressed sensing can reconstruct enterprise debt network links with less prior information. Simulation results show that the compressed sensing method can reconstruct over 70% of links in a real debt network and find almost all unknown links, and the error is controlled within 2%. The key of enterprise debt network reconstruction is the sparsity of column vector in network link matrix. Since an enterprise usually has debt relationships with a small amount of other enterprises, which satisfies the requirement of sparsity. Therefore, we can use compressed sensing method to realize the high-precision reconstruction of enterprise debt network.

## Supplementary Information

Supplementary Information.

## Data Availability

All data generated or analysed during this study are included in this published article and its supplementary information files.
